# Application of the patient-reported outcome-based postoperative symptom management model in lung cancer: a multicenter randomized controlled trial protocol

**DOI:** 10.1186/s13063-024-07963-8

**Published:** 2024-02-17

**Authors:** Ying Liang, Pengyu Jing, Zhongping Gu, Lei Shang, Peng Ge, Yong Zhang, Lv Wang, Chun Qiu, Ximing Zhu, Zhijun Tan

**Affiliations:** 1 Department of Health Statistics, Ministry of Education Key Lab of Hazard Assessment and Control in Special Operational Environment, Airforce Military Medical University (Fourth Military Medical University), Xi’an, 710032 Shaanxi Province China; 2 Department of Thoracic Surgery, Tangdu Hospital, Xi’an, 710000 Shaanxi Province China; 3 Department of Thoracic Surgery, The Second Affiliated Hospital of Xi’an Medical College, Xi’an, 710038 Shaanxi Province China; 4 Department of Thoracic Surgery, The Affiliated Hospital of Shaanxi University of Traditional Chinese Medicine, XianYang, 712000 Shaanxi Province China; 5 Department of Thoracic Surgery, Daxing Hospital, Xi’an, 710000 Shaanxi Province China; 6 Department of cerebral Surgery, Tangdu Hospital, Xi’an, Shaanxi Province 710000 China

**Keywords:** Lung cancer, Patient-reported outcome, Symptom management, Randomized controlled trial

## Abstract

**Introduction:**

Lung cancer is the most common cancer in China, with the highest mortality rate. Surgery is the primary treatment for early lung cancer. However, patients with lung cancer have a heavy burden of symptoms within 3 months after surgery, which seriously affects their quality of life (QOL). The symptom management model based on the patient-reported outcome (PRO) is considered the best caregiving model. The clinical evidence about the symptom management of lung cancer within 3 months after the operation is very limited. Herein, we propose a randomized controlled trial to evaluate the PRO score-based monitoring and alert system for follow-up on psychological and physiological symptoms of lung cancer patients within 3 months after surgery and further investigate the effect of intervention measures based on this PRO score-based system.

**Methods and analysis:**

This multicenter, open-label, randomized, parallel superiority trial will be conducted at four hospitals in China. A total of 440 lung cancer patients will be recruited in this study, who will be randomly assigned to the intervention group or the control group in a ratio of 1:1. Any of the target symptoms reaches the preset threshold (score ≥ 4), the patients will accept the symptom management advices based on the PRO. The patients in the control group will follow the current standard procedure of symptom management. The symptom management system is an electronic management system based on WeChat mini programs. All patients will be evaluated for symptoms through the lung cancer module of the MDASI lung cancer-specific scale on the day before surgery, days 1, 3, 5, and 7 after surgery, and once a week during the 12-week post-discharge period. Simultaneously, the EORTC QLQ-C30 scale will be used to evaluate patients’ quality of life at baseline and the fourth and twelfth week after the surgery. The mean number of symptom threshold events of the intervention and the control groups were compared by *t*-test, and the changes of PRO were compared by a mixed effect model. The primary endpoint has been set as the 12-week post-discharge period.

**Discussion:**

This study will test the feasibility of the symptom management system based on the mobile social media applet in postoperative caregiving and the efficacy of psychiatrist-assisted treatment and provide evidence in managing the symptoms of patients in the medium and long term.

**Trials registration:**

Trials registration number: ChiCTR 2200058876, Registered 18 April 2022

**Supplementary Information:**

The online version contains supplementary material available at 10.1186/s13063-024-07963-8.

## Background

Lung cancer is the malignant tumor with the highest morbidity and mortality worldwide. The incidence of lung cancer in China is more than 1/3 of the world’s reported cases [1]. With the increase in early lung cancer surgery, postoperative management and quality of survival have gradually become the focus of caregiving [2, 3]. Since the burden of postoperative symptoms and the incidence of postoperative complications are overwhelmingly high within 3 months after lung surgery, their quality of life (QOL) can be severely deteriorated by severe pain, cough, shortness of breath, fatigue, sleep disorders, and other physiological symptoms [4].

In addition to physiological symptoms, almost all cancer patients will also experience severe psychological disturbance from sorrow and distress. Related lung cancer studies have shown that the sadness and distress of patients with lung cancer are about 40%. Chinese patients are close to this level, and the emotions and symptoms are closely related to the quality of life [4–8]. Studies have shown that lung cancer patients held the highest incidence of psychological stress among all cancers [5, 9]. The symptoms of fatigue, pain, sadness, uneasiness, and distress, separately or corporately, constitute the most common psychological features within 3 months after the lung operation. Thus, the caregiving of patients with lung cancer needs to underpin the management of physiological symptoms and commit to tuning down the extent of distress and sad emotional symptoms. Practically, all the symptoms of postoperative lung cancer patients can be considered the parameters for further caregiving [10].

**Fig. 1:**
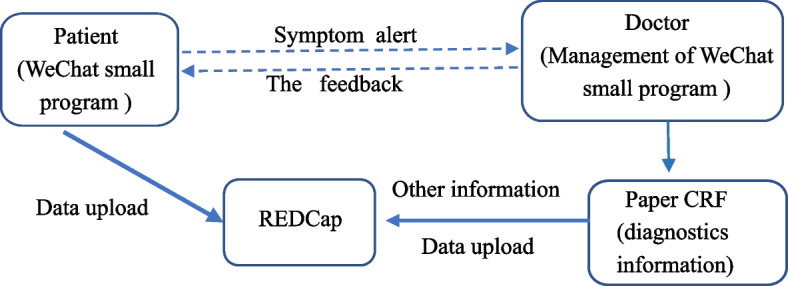
Process of symptom monitoring, early warning, feedback, and data collection; REDCap, research electronic data capture. CRF, case report form

With the concept of “patient-centered care,” the patient-reported outcome (PRO), which is directly reported by the patient and details the patient’s health, functional status, and treatment experience with the exclusion of the interpretation from any other personnel, has been popularized in the caregiving process to abide with the “biological-psychological-social” medical model (Fig. 1) [11, 12]. Since the report directly from patients cancels the interferences and noises from the ones who are not the patients, PRO can mirror the real-time changes and manifestations of the patient’s symptoms, which, on the one hand, can more accurately guide the clinicians to choose more accurate intervention, on the other hand, can continuously evolve the treatment methods [13, 14].

Symptom management has been entrenched as the cornerstone of medical care. The symptom management based on PRO improved patients’ QOL, reached patient satisfaction, promoted the corporate decision-making of caregiver and patient, and benefited the clinical prognosis [14]. The previous studies on the efficacy of PRO-based symptom management only focused on single symptoms or were observational research [15–17], which had shown that patients with advanced chemotherapy could extend the survival time by 5.2 months(*P =* 0.03) with the PRO-based active symptoms monitoring in comparing with traditional passive monitoring. In addition, a randomized controlled study reported that physiological symptom monitoring within 1 month after lung cancer surgery made the threshold events and incidence of complications in the intervention group lower than those in the control group (0 vs. 2, *P* = 0.007; 21.5% vs. 40.6%, *P* = 0.019, respectively) [18, 19]. However, the study did not further address whether monitoring and managing psychological symptoms during the surgery could benefit the patients by prolonging survival time [20–25].

Although the application of PROs has gradually been promoted in the surgical treatment environment, very few clinical trials and studies were conducted on postoperative symptoms of lung cancer, containing physiological and psychological symptoms. Besides, the limitations of those previous studies included the following: (1) most were observational researches; (2) all of them focused only on managing physiological symptoms such as cough, pain, shortness of breath, and lack of patients with psychological symptoms such as sadness and distress; (3) all had an insufficient assessment on the symptoms; (4) there are even fewer studies on the Chinese population, which undermine paradigm change in the caregiving of lung cancer patients.

WeChat platform has become China’s leading online social network [26], providing real-time online person-to-person and group communication. So, we have designed this randomized controlled trial (RCT) to improve the effectiveness of postoperative caregiving for patients with lung cancer-based physiological (pain, cough, fatigue, sleep disorders, and shortness of breath) and psychological symptoms (sadness and distress) through the WeChat application, which can carry out the real-time monitoring of the early red-flag signs from various postoperative symptoms of the patients, and conveniently facilitate the clinicians to prescribe post-surgical palliative interventions (The operating mechanism is shown in Fig. 1). The PRO-based intervention methods will be used to manage patients with moderate and severe target symptoms in the intervention group, while current standard nursing methods will be used to care the patients in the control group. This trial will introduce a novel, safe methodology for managing medium and long-term symptoms in lung cancer patients. We will use the MD Anderson Symptom Inventory for lung cancer (MDASI-LC) [27] on the patients within 3 months before and after surgery to record the monitoring of target symptoms as well as related effects on the function of lung cancer patients. At the same time, the EORTC Core quality of life questionnaire (EORTC QLQ-C30) [28] was attempted to evaluate the quality of life of patients with lung cancer within 3 months before and after the lung operation. As a result, the aim of this study is to compare on the mean number of occurrences of target symptoms and survival quality of the trial participants who received PRO-based management to those of the patients undergoing the routine management.

## Methods

### Trial design

This multicenter, open-label, randomized, parallel-group, superiority trial will be conducted at four tertiary hospitals in two cities of Western China, including Tangdu Hospital, Daxing Hospital, the Affiliated Hospital of Shaanxi University of Traditional Chinese Medicine, and the Second Affiliated Hospital of Xi’an Medical College. The total number of lung cancer operations is approximately 5000 in these four hospitals yearly.

**Fig. 2:**
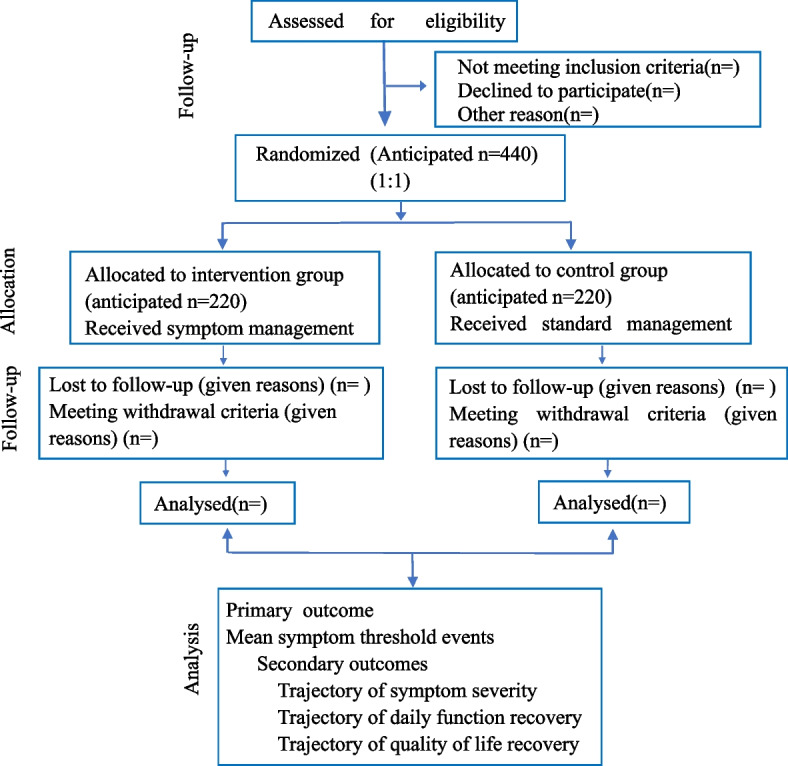
Flow chart of this parallel-group randomized trial

Participants who fulfill eligibility criteria will be randomly assigned with a 1:1 ratio to either a control group with current standard management or an intervention group with new management methods designed by the lung cancer specialist and psychological consultants from the four hospitals. This protocol abided by the standard protocol for clinical trials [29], and the flow chart of this trial is shown in Fig. 2.

### Randomization and blinding

The stratified block randomization method will be used for this trial. The third-party statisticians will use the statistical software SAS9.4 PROC PLAN to generate random numbers and randomize the participating patients. Participants will be stratified by research center, and the appropriate segment lengths will be selected. Allocation of participants will be concealed using envelopes with consecutive numbers. The randomized numbers will be segmented, retained, and managed by the third party who will not be involved in the data collection. Each center assigned its participants according to the estimated number of participants and the enrollment sequence of each participant in that center. The random grouping table is pre-implanted into the WeChat applet. It will then be allocated to the intervention group or control group with a 1:1 ratio. Due to the nature of the intervention, the participating patients, specialist cannot be blinded. In order to reduce measurement bias, all personnel who assist in data collection and data analysts (the third party) will be blinded. As a result, unblinding will not occur in this trial.

### Study population

The recruitment will be conducted by the participating clinicians before surgery. Eligible patients should meet all inclusion criteria and should not meet any of the exclusion criteria. The inclusion criteria for participating in this trial include (1) the age between 18 and 75, (2) an imaging study showing that lung cancer is highly suspicious, (3) Eastern Cooperative Oncology Group (ECOG) PS 0–2 points [30], (4) normal primary organ function, (5) resectability with the estimated survival time beyond 6 months, (6) voluntariness to join the study with signed informed consent for participating the trial, and (7) compliance and cooperation during the trial.

The exclusion criteria were also settled as follows: (1) history of neoadjuvant therapy, (2) suffering from other malignant tumors, (3) inability to understand the research requirements, (4) existence of concomitant diseases or any other conditions that may confuse the results of the study or affect the subjects to complete the study, (5) history of psychotropic substance, alcoholism, or drug abuse, (6) possibility that may damage the subject or cause the subject to be unable to meet or perform the research requirements.

### Sample size calculation

The primary endpoint of this study was the mean number of symptom threshold events, which is calculated by averaging the number of target symptom threshold events per patient at each time point. The minimum important difference (MID) has been defined as the smallest difference in the scores of the PRO measure that patients perceive as beneficial or harmful and would lead the clinician to consider a change in treatment. The literature recommended that the minimum difference between the two groups should be ≥ 0.3 sd based on the clinical treatment comparison efficacy measured by PRO instruments [31]. To meet the MID (0.3 sd) with the PRO instrument for mean symptom threshold events, the sample size required for each group was 176 when rejecting the null hypothesis (difference between the two groups < 0.3 SD). A total of 352 samples are required in this study. According to the previous pre-investigation experience, the loss rate of follow-up and withdrawal was 20%; 220 patients should be the adequate number of participating patients for each group (176/0.8). Because this trial is an intervention study, the follow-up time is 3 months after major lung operations. The sample size calculation is based on the independent sample Student’s *t*-test, using a two-tailed test (*α* = 0.05, 1 − *β* = 0.8).

### Screening period

Before enrolling patients, researchers in each research center will receive standard operating procedure (SOP) training on research implementation methods and data collection. During the screening period, the data on participants’ demographics, physical examinations, current medical conditions, and history of comorbidities will be collected. Thoracic surgeons will mainly conduct the screening of participants. The surgeons will select patients who underwent surgery in the trial according to the inclusion criteria. Then, the clinical research associate will register all eligible patients for the trial. Patients who meet the screening criteria will be given a screening number. Then, if patients agree to participate in this trial, they will be given a subject ID and fill in MDASI-LC QLQ-C30 before surgery. Results in the screening period will be considered baseline data and will not be required to be repeated.

Surgeons who screen patients will be paid to increase their enthusiasm for work, and we will also invite as many surgeons in other inpatient areas as possible to participate in the trial to meet the samples required for the study.

### Intervention period

The questionnaire and parameters that will be measured to assess symptoms and life quality at each visit point are shown in Table 1. The participants will answer the MDASI-LC (ePRO) questionnaire through a WeChat applet within 1 week before surgery, the first, third, fifth, and seventh days after surgery, and every week after discharge till the 12th week. Participants can fill in the ePRO questionnaire by scanning the two-dimensional code and logging into their personal account after receiving the reminder message to fill in the WeChat applet. Moreover, the paper version of QLQ-C30 filled by the same participants within 1–2 days before the operation, 4 weeks, and 12 weeks after the operation will be submitted to the clinicians when the review process is completed. When one or more target symptoms (pain, cough, fatigue, sleep disorder, shortness of breath, sadness, and distress) occur and the score reaches the preset intervention threshold (above moderate, score ≥ 4) [32], the participants in intervention group will receive the standardized treatment suggestions in WeChat applet. At the same time, the participating specialists (thoracic surgeons) will also receive early alerting messages on their mobile phones to inform them to contact the patient through WeChat or phone within 24 h to provide the patient with management advices base on ePRO score, including, if not at all, consultation, education, drug guidance, and evaluation on the necessity of the trip to emergence, clinic, or hospital. In addition to the target symptoms, if the participants have other novel symptoms or their ePRO exceeds the threshold, standard treatment suggestions designed by the lung cancer specialist will emerge on the WeChat ePRO interactive interface [33–38]. The psychiatrists focus on the psychological symptoms (sadness and distress) of all patients in the intervention group. If the score is between 4 and 6 points, guidance to manage the psychological symptom will be given to the patients by the general specialist, while if the score exceeds 6 points, the patients will receive a telephone consultation with the psychiatrists. The measure used for symptom relief in the intervention group will comply with the latest guidelines and follow SOP manuals in all centers. In addition, the participating patients are not informed of the threshold level. Specialist will not receive warning messages from the PRO data of control group patients and will not access the patient data in the control group. During the trial, each center will provide the participating patients with on-site monitoring visits, telephone monitoring, and online or on-site guidance on managing the symptoms. If the patients concern their symptoms, they will decide whether to seek medical help based on their self-evaluation of their symptoms. The participating patients will be educated on how to seek medical help for severe symptoms through usual channels. All participants will be provided free long-term medical consultation via WeChat, which may promote participation and compliance.

**Table 1:** Study plan detailing the procedures

**Study period**	**Screening**		**Intervention period**						
Visit	V1	V2	V3 (post-operation)	V4	V5	V7	V8	V9	
Visit day	− 1 (± 2 days)	Randomization/baseline	1 (± 1 day)	3 (± 1 day)	5 (± 1 day)	7 (± 1 day)	14 (± 3 days)	22 (± 3 days)	
**Screening/demography**									
Written informed consent	√								
Inclusion/exclusion criteria	√								
Demographics	√								
Physical examination, height, and weight	√								
Medical/current conditions	√								
ECOG (Eastern Cooperative Oncology Group)	√								
CCI (Charlson Comorbidity Index)							√		
**Intervention**									
Consultation, education, drug guidance, telephone consultation with the psychiatrists			√	√	√	√	√	√	
MDASI-LC (ePRO)		√	√	√	√	√	√	√	
QLQ-C30 (paper)		√							
Adverse events	√	√	√	√	√	√	√	√	
**Clinical characteristics**									
Surgical procedure, surgical approach, extent of surgery, stage, postoperative length of stay, postoperative chemotherapy							√		
**Study period**	**Intervention period**								
Visit	V10	V11	V12	V13	V14	V15	V16	V17	V18
Study day	29 (± 3 days)	36 (± 3 days)	43 (± 3 days)	50 (± 3 days)	57 (± 3 days)	64 (± 3 days)	71 (± 3 days)	78 (± 3 days)	86 (± 3 days)
**Screening/demography/baseline**									
Written informed consent									
Inclusion/exclusion criteria									
Demographics									
Physical examination, height, and weight									
Medical/current conditions									
ECOG (Eastern Cooperative Oncology Group)									
CCI (Charlson Comorbidity Index)									
**Intervention**									
Consultation, education, drug guidance	√	√	√	√	√	√	√	√	√
MDASI-LC (ePRO)	√	√	√	√	√	√	√	√	√
QLQ-C30 (paper)	√								√
Adverse events	√	√	√	√	√	√	√	√	√
**Clinical characteristics**									
Surgical procedure, surgical approach, extent of surgery, stage, postoperative length of stay, postoperative chemotherapy									

Interventions are based on participants’ ePRO scores, and if there is a target symptom score ≥ 4, there will be corresponding treatment

The adherence to the intervention measures will be evaluated at every given time. If the participants fail to comply with the intervention measures as required more than two times, it will be considered a severe violation of the study protocol and removed from the trial. If the participants have serious complications after the operation and cannot complete the PRO questionnaire with poor compliance or deliberately miss the questionnaire filling or request to withdraw, they will be stopped from further collecting the relevant data.

### Control group

The patients in the control group do not receive any specific guidance on related symptoms except by completing the same questionnaire as the intervention group. The postoperative management of patients follows the current standard management in the hospital and after discharge. Following discharge, all the participants follow-up at the same time as the intervention group. At the same time, we encourage participants to actively seek medical help if necessary.

### Statistical analysis

The primary and secondary outcomes will be analyzed based on the protocol set. To be included in the analyses, a participant must provide MDASI-LC data from the baseline and at least two additional time points, QLQ-C30 data from the baseline, and at least one postoperative time point. All outcomes will also be analyzed based on full analysis set. The analysis will be carried out on the thoroughly collected data with the definition of statistical significance as *P* ≤ 0.05. Classification variables will be expressed in frequency or proportion and compared between groups with the *χ*^2^ test. Continuous variables will be represented by $\overline{x}\pm s$ or *M*(*P*_25_, *P*_75_) and compared between groups using Student’s test or Wilcoxon rank sum test. The changes in PROs in the intervention and control groups will be compared using the generalized mixed-effect model. Missing data will be processed by multiple interpolations. Subgroup analysis of patients undergoing postoperative chemotherapy. Sensitivity analysis is mainly to compare the data without missing data to the data set with multiple imputed data.

## Results and measurements

### Primary outcomes

The main PRO tools in this study include MDASI-LC and EORTC QLQ-C30. MDASI-LC covers 16 lung cancer- and treatment-related symptoms as well as six disturbances to everyday life caused by symptoms and can be completed in five minutes. All items were scored on a scale of 0–10, with 0 as “no symptoms” or “no impact on life” and 10 as “the most serious level” or “complete impact on life.” Besides, the patients can have 24 h as the recall period before filling in MDASI-LC. Comparably, EORTC QLQ-C30 lays out 30 items, incorporating five functional dimensions, three symptom dimensions, six single symptom dimensions, and two total health status items. These can be done in 5 to 8 min with a recall period of 1 week. EORTC QLQ-C30 scores items 1–27 by the range of 1–4 points, with 1 as “no symptoms” or “no impact on life” and 4 as “the most serious level” or “complete impact on life.” Additionally, items 28 and 29 focus on health status assessment and can be scored by 1 to 7, with 1 as very poor and 7 as very good. For the evaluation, the severity of symptoms and function deterioration increases along the increments of the scores. Conversely, the higher the health score, the better the quality of life. Both scales have been widely used in China’s clinical setting with good reliability and validity [39, 40]. The main measurement indicators and follow-up schedule are shown in Table 1. The primary outcome of this study is the mean number of target symptom threshold events with discharge 12 weeks, which include pain, cough, fatigue, sleep disorders, shortness of breath, sadness and distress. According to published literature, patients with a symptom score of 4–6 can be classified as moderate and ≥ 7 as severe [33]. Thus, we presented the score of 4 as the threshold for intervention and the targeted symptom score of ≥ 4 as a threshold event in this trial. A score of 1 can be given if one of the target symptoms is recorded to be ≥ 4 in one follow-up and 3 if three target symptoms are graded as ≥ 4.

### Secondary outcomes

The secondary outcomes of this study include the changes in PROs (symptom severity, daily function, and quality of life) and follow-up rates after discharge. The trajectory for PROs is defined as the average score of symptom severity for seven targeted symptoms and the average score of six MDASI-LC daily functional intervention items from baseline to 12 weeks after discharge. The trajectory for quality of life comes from the quality of life scale (EORTC QLQ-C30), including the measurement results at baseline and the 4th and 12th weeks after discharge.

### Other data

The specialist workload, the acceptability of the trial interventions, and the satisfaction of patients will be assessed through surveys and interviews. Experts’ workload is mainly determined by the average amount of time spent on each patient’s work through interviews, while experts assess the number of patients managed within a particular time. The acceptability and satisfaction of patients are mainly investigated through questionnaires, including the convenience and satisfaction of using WeChat mini-programs and satisfaction with expert postoperative guidance, among others. Demographic and clinicopathological features, follow-up information, and adverse events of interventions will also be obtained. All adverse events will be evaluated and managed by the thoracic surgeon.

### Patient and public participation statement

Researchers carried out the design, recruitment, and implementation of this study. All the participants are prevented from obtaining the research plan and the results from the ongoing trial. The participants can obtain the final results of this study through our future articles.

### Ethics and communication

All recruited patients must sign the written informed consent for the trial participation before the trial starts. Any subsequent amendments to the protocol will be submitted for further review and approval. The sub-center qualification will be approved and accredited by the hospital-specific ethics committee.

### Data collection, management, and quality control

All participants will be recognized only by their subject ID. MDASI-LC data will be gathered into digital questionnaires through the WeChat applet, which will then be connected to the REDCap platform [41]. The collected paper questionnaire data will be entered into the RedCap platform online by the research assistant of each center. The data of this study adopts the REDCap data collection and management platform built by Xi’an Jiaotong University. All the information of participants will be kept strictly confidential and will be accessed only by members of the trial team or ethics committee. Data in paper files and from WeChat applet will be safely stored in online REDCap and inspected by a third party who is not involved in this study. All the participants will be blinded from obtaining the research plan and the results from the ongoing trial. All the participating patients will take on the WeChat platform to fill in the MDASI-LC questionnaire through an applet designed for this trial. To ensure the accuracy of the data, every participant receives training on how to use the procedure before formal filling. Participants will usually fill in electronic and paper questionnaires by themselves. If participants without smartphones or not using WeChat be addressed, or encounter difficulties filling out the electronic questionnaire, they will be assisted by a data collection staff or their family members who are familiar with the platform and applet to complete the questionnaire. During hospitalization, each patient receives a bedside reminder (one day after the operation) and an electronic reminder twice daily to fill in the MDASI-LC on the scheduled date. After discharge, if the participating patient does not fill in the MDASI-LC by 20 o’clock, they will be personally reminded through the phone or WeChat call by the project management personnel. The paper questionnaires of EORTC QLQ-C30 will be filled by the participants, while other diagnosis-related information will be manually collected and filled in paper table by the trial staff; then, those data will be entered into RedCap. All data on the paper questionnaire will be repeatedly checked by the data entry clerk before and after data are input into the Redcap. All the data will then be checked by the outcome assessors for any errors and missing values before analysis. Each participant in data management and participating doctors have signed a data confidentiality agreement.

### Data monitoring and interim analysis

A data monitoring committee (DMC) will be established on a panel of a clinician, a statistician, and the secretary of the Tangdu ethics committee. DMC members will regularly and independently monitor the safety, validity, and integrity of the randomized parallel control study once every 3 months. DMC will meet twice a year or more if needed. No interim analysis will be conducted because this trial is low-risk and short-term.

## Discussion

**Fig. 3:**
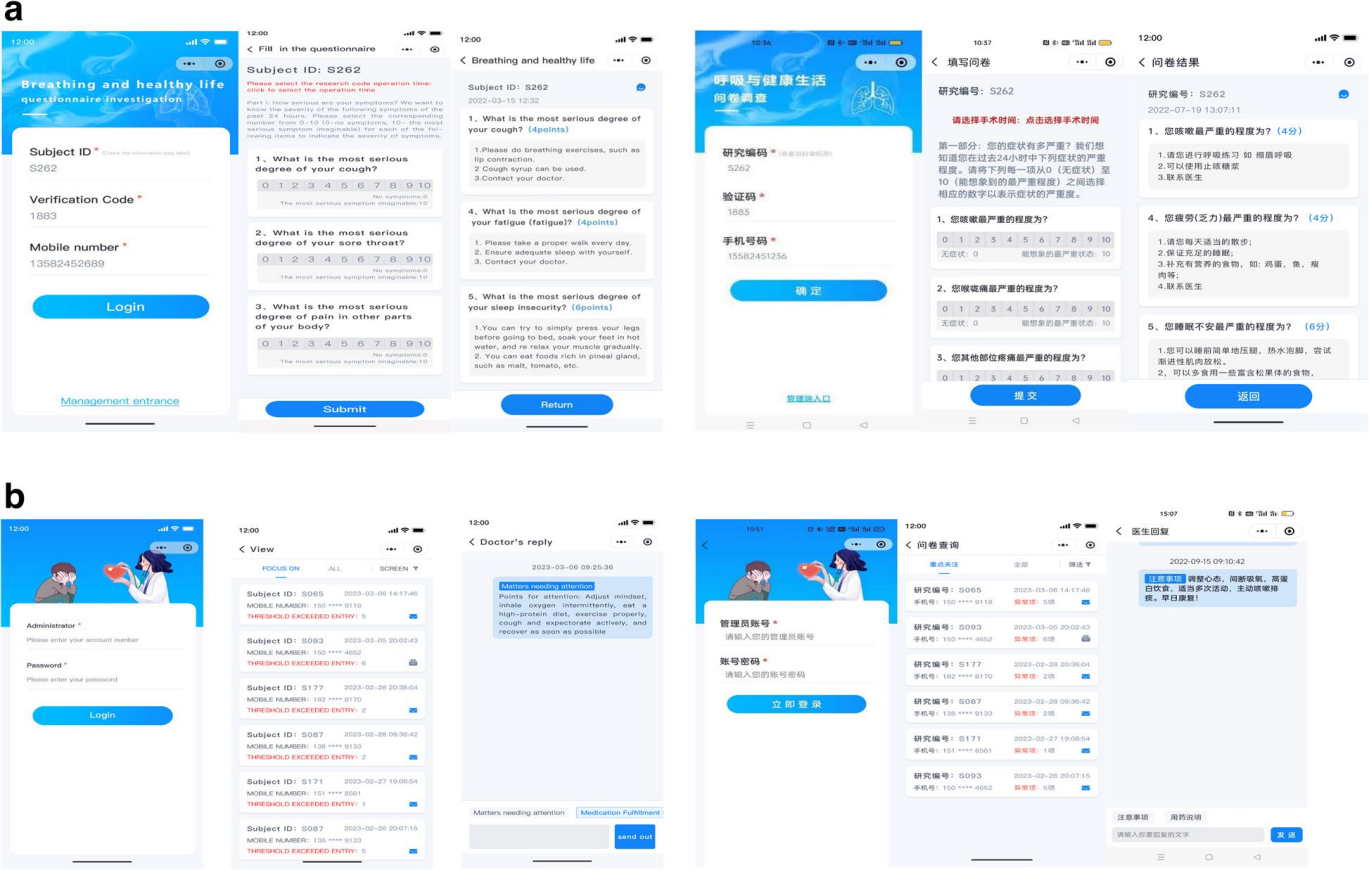
**a** Pop-up window of participant questionnaire filling and standardized symptom management opinions of intervention group (English and Chinese versions). **b** Participant management and interaction window (English and Chinese versions)

This study focused on managing early, medium, and long-term postoperative symptoms after lung cancer surgery. The potential impacts of the findings from this trial possibly include the following: (1) determining whether the PRO-based physiological and psychological symptom management is superior to routine management and whether it is superior to people who only carry out physiological symptom management, (2) determining whether active physiological and psychological symptom management can effectively reduce the burden of symptoms and improve the quality of life of the patients with major lung surgeries, (3) to establish a foundation for future research on whether long-term postoperative symptom management (physiological and psychological symptoms) can improve the survival rate, (4) to investigate whether the postoperative management of lung cancer patients based on WeChat and the intervention of psychiatrists is feasible and acceptable in the actual clinical practice in China, (5) to open a niche for the research on the necessity of psychiatrists in the management of lung cancer patients’ symptoms. The psychological and emotional changes of cancer patients may profoundly affect the prognosis and quality of life of patients. Through the WeChat or phone-based (Fig. 3) guidance, this study has been anticipated to effectively relieve the physiological and psychological pressures on patients, facilitate doctor-patient communication, and provide a new approach for the follow-up and guidance of future lung cancer patients in managing postoperative symptoms.

This experiment has some limitations. First, the trial will be conducted in China’s well-resourced tertiary hospitals with the participation of psychiatrists, which limits the versatility of hospitals that lack psychologists. Second, the inclusion and exclusion criteria are stringent. For example, patients who do not use smartphones may be excluded, resulting in selection bias. Third, the participating patients and specialists who provide interventions cannot be blinded, leading to measurement bias. Fourth, the patients may take more than 15 min to complete the questionnaire, increasing the burden on patients.

### Strengths and limitations of this study

This multicenter randomized controlled trial will be conducted in four tertiary hospitals in China, comparing the effects of patient-reported versus standard postoperative symptom management in patients with lung cancer. In patient-reported management, the lung cancer-specific scale MDASI-LC will be integrated into an online questionnaire survey in the early, medium, and long-term management after the operation, which covers the clinical information of preoperative baseline, postoperative hospitalization, 1, 3, 5, and 7 days after the operation, and once a week after discharge until 12 weeks. Moreover, EORTC QLQ-C30 was used to investigate the quality of life at baseline and 4 and 12 weeks after the operation. Various postoperative adverse events or clinical issues of the patients will be instantly managed through the WeChat platform. Essentially, this trial will monitor and manage the physiological symptoms of the disease and the emotional symptoms of the patients. However, due to the demand for the participation of psychiatrists in this new caregiving model, the methodology and outcome of this trial may be limited by the promotion and application of some hospitals.

In conclusion, as a randomized controlled trial, this study will test the feasibility of the symptom management system based on the WeChat applet in postoperative caregiving and the efficacy of psychiatrist-assisted treatment and provide evidence in managing patients’ symptoms in the medium and long term.

## Trial status

The study began recruitment on 20 April 2022 and will continue until the last patient’s visit (anticipated 30 June 2023). Current protocol: version 1.2 (20.08.2021). Trial registration number: ChiCTR 2200058876, Registered 18 April 2022. The name of the registry is Chinese Clinical Trial Registry and the URL of the trial registry record is https://www.chictr.org.cn/.

### Supplementary Information

**Additional file 1.**

## Data Availability

The raw data are available upon reasonable requests to the corresponding authors.

## Abbreviations

QOL: Quality of life
PRO: Patient-reported outcome
RCT: Randomized controlled trial
MDASI-LC: MD Anderson symptom inventory for lung cancer
EORTC QLQ-C30: EORTC core quality of life questionnaire
ECOG: Eastern cooperative oncology group
MID: Minimum important difference
SOP: Standard operating procedure
DMC: Data monitoring committee
CCI: Charlson comorbidity index

## References

[1] Chen W, Zheng R, Zeng H, Zhang S (2015). Epidemiology of lung cancer in China. Thorac Cancer..

[2] Yun YH, Kim YA, Sim JA, Yun YH, Kim YA, Sim JA, Shin AS, Chang YJ, Lee J, Kim MS, Shim YM, Zo JL (2016). Prognostic value of quality of life score in disease-free survivors of surgically-treated lung cancer. BMC Cancer..

[3] Sim JA, Kim YA, Kim JH, Sim JA, Kim YA, Kim JH, Lee JM, Kim MS, Shim YM, Zo JI, Yun YH (2020). The major effects of health-related quality of life on 5-year survival prediction among lung cancer survivors: applications of machine learning. Sci Rep..

[4] Si C, Chuntao L, Yulan O (2021). Investigation of symptom groups in patients with lung cancer 3 months after operation. Mod Med Health..

[5] Linares-Moya M, Rodríguez-Torres J, Heredia-Ciuró A, Granados-Santiago M, López-López L, Quero-Valenzuela F, Valenza MC (2022). Psychological distress prior to surgery is related to symptom burden and health status in lung cancer survivors. Support Care Cancer..

[6] Deng Y, Peng L, Li N, Zhai Z, Xiang D, Ye X, Hu J, Zheng Y, Yao J, Wang S, Wei B, Xu P, Zhang D, Chen T, Dai Z (2021). Tracheal, bronchus, and lung cancer burden and related risk factors in the United States and China. Am J Transl Res..

[7] Morrison EJ, Novotny PJ, Sloan JA, Yang P, Patten CA, Ruddy KJ, Clark MM (2017). Emotional problems, quality of life, and symptom burden in patients with lung cancer. Clin Lung Cancer..

[8] Wu CM, Zhou HL, Jiang YH, Wang YQ, Liao J, Yang F, Feng YQ, Wei X, Li Q, Wei D, Shi QL (2021). Preoperative self-reported symptom burden and quality of life of lung cancer patients: a cross-sectional study. J Cancer Control Treat..

[9] Lin S, Chen Y, Yang L, Zhou J (2013). Pain, fatigue, disturbed sleep and distress comprised a symptom cluster that related to quality of life and functional status of lung cancer surgery patients. J Clin Nurs..

[10] Koch M, Gräfenstein L, Karnosky J, Schulz C, Koller M (2021). Psychosocial burden and quality of life of lung cancer patients: results of the EORTC QLQ-C30/QLQ-LC29 questionnaire and Hornheide screening instrument. Cancer Manag Res..

[11] Weldring T, Smith SM (2013). Patient-reported outcomes (PROs) and patient-reported outcome measures (PROMs). Health Serv Insights..

[12] Rogge A, Fischer F, Otto L, Rose M (2022). Empirische Erfassung patient*innenberichteter Merkmale: PROMs und PREMs. Anasthesiol Intensivmed Notfallmed Schmerzther..

[13] Morton RL, Lioufas N, Dansie K, Palmer SC, Jose MD, Raj R, Salmon A, Sypek M, Tong A, Ludlow M, Boudville N, McDonald S (2020). Use of patient-reported outcome measures and patient-reported experience measures in renal units in Australia and New Zealand: a cross-sectional survey study. Nephrology (Carlton)..

[14] Duncanson E, Bennett PN, Viecelli A, Dansie K, Handke W, Tong A, Palmer S, Jesudason S, McDonald SP, Morton RL (2020). Feasibility and acceptability of e-PROMs data capture and feedback among patients receiving haemodialysis in the Symptom monitoring WIth Feedback Trial (SWIFT) pilot: protocol for a qualitative study in Australia. BMJ Open..

[15] Mendoza TR, Kehl KL, Bamidele O, Williams LA, Shi Q, Cleeland CS, Simon G (2019). Assessment of baseline symptom burden in treatment-naïve patients with lung cancer: an observational study. Support Care Cancer..

[16] Wei X, Yu H, Dai W, Xu W, Yu Q, Pu Y, Wang Y, Liao J, Li Q, Shi Q (2022). Discrepancy in the perception of symptoms among patients and healthcare providers after lung cancer surgery. Support Care Cancer..

[17] Wong ML, Paul SM, Cooper BA, Dunn LB, Hammer MJ, Conley YP, Wright F, Levine JD, Walter LC, Cartwright F, Miaskowski C (2017). Predictors of the multidimensional symptom experience of lung cancer patients receiving chemotherapy. Support Care Cancer..

[18] Basch E, Deal AM, Dueck AC, Scher HI, Kris MG, Hudis C, Schrag D (2017). Overall survival results of a trial assessing patient-reported outcomes for symptom monitoring during routine cancer treatment. JAMA..

[19] Dai W, Feng W, Zhang Y, Wang XS, Liu Y, Pompili C, Xu W, Xie S, Wang Y, Liao J, Wei X, Xiang R, Hu B, Tian B, Yang X, Wang X, Xiao P, Lai Q, Wang X, Cao B, Wang Q, Liu F, Liu X, Xie T, Yang X, Zhuang X, Wu Z, Che G, Li Q, Shi Q (2022). Patient-reported outcome-based symptom management versus usual care after lung cancer surgery: a multicenter randomized controlled trial. J Clin Oncol..

[20] Dobrozsi S, Panepinto J (2015). Patient-reported outcomes in clinical practice. Hematology Am Soc Hematol Educ Program..

[21] Johnston BC, Patrick DL, Devji T, Maxwell LJ, Bingham CO, Beaton D, Boers M, Briel M, Busse JW, Carrasco-Labra A, Christensen R, da Costa BR, El Dib R, Lyddiatt A, Ostelo RW, Shea B, Singh J, Terwee CB, Williamson PR, Gagnier JJ, Tugwell P, Guyatt GH (2023). Chapter 18: Patient-reported outcomes.

[22] Li Y, Zheng E, Mei Y, Yuanyuan Y (2023). A symptom survey of patients after thoracoscopic lung cancer resection based on patient reported outcomes. Chinese. J Thorac Cardiovasc Surg..

[23] Brønserud MM, Iachina M, Green A, Groenvold M, Dørflinger L, Jakobsen E (2019). Patient-reported outcomes (PROs) in lung cancer: experiences from a nationwide feasibility study. Lung Cancer..

[24] Brønserud MM, Iachina M, Green A, Groenvold M, Jakobsen E (2019). Patient reported outcome data as performance indicators in surgically treated lung cancer patients. Lung Cancer..

[25] Steffen McLouth LE, Lycan TW, Levine BJ, Gabbard J, Ruiz J, Farris M, Grant SC, Pajewski NM, Weaver KE, Petty WJ (2020). Patient-reported outcomes from patients receiving immunotherapy or chemoimmunotherapy for metastatic non-small-cell lung cancer in clinical practice. Clin Lung Cancer..

[26] Montag C, Becker B, Gan C (2018). The multipurpose application WeChat: a review on recent research. Front Psychol..

[27] Mendoza TR, Wang XS, Lu C, Palos GR, Liao Z, Mobley GM, Kapoor S, Cleeland CS (2011). Measuring the symptom burden of lung cancer: the validity and utility of the lung cancer module of the MD Anderson Symptom Inventory. Oncologist..

[28] Snyder CF, Blackford AL, Okuyama T, Akechi T, Yamashita H, Toyama T, Carducci MA, Wu AW (2013). Using the EORTC-QLQ-C30 in clinical practice for patient management: identifying scores requiring a clinician’s attention. Qual Life Res..

[29] Chan A-W, Tetzlaff JM, Gøtzsche PC, Altman DG, Mann H, Berlin J, Dickersin K, Hróbjartsson A, Schulz KF, Parulekar WR, Krleža-Jerić K, Laupacis A, Moher D (2013). SPIRIT 2013 explanation and elaboration: guidance for protocols of clinical trials. BMJ..

[30] Azam F, Latif MF, Farooq A, Tirmazy SH, AlShahrani S, Bashir S, Bukhari N (2019). Performance status assessment by using ECOG (Eastern Cooperative Oncology Group) score for cancer patients by oncology healthcare professionals. Case Rep Oncol..

[31] Revicki D, Hays RD, Cella D, Sloan J (2008). Recommended methods for determining responsiveness and minimally important differences for patient-reported outcomes. J Clin Epidemiol.

[32] Zhirong Y, Ting Z, Jun Z, Rui L, Lei L, Heli C (2021). A study of postoperative symptom recovery in patients with ovarian cancer based on self-reported outcomes. Cancer Prev Treat..

[33] Bower JE, Bak K, Berger A, Breitbart W, Escalante CP, Ganz PA, Schnipper HH, Lacchetti C, Ligibel JA, Lyman GH, Ogaily MS, Pirl WF, Jacobsen PB (2014). Screening, assessment, and management of fatigue in adult survivors of cancer: an American Society of Clinical oncology clinical practice guideline adaptation. J Clin Oncol..

[34] Zhang L, Liu X, Tong F, Zou R, Peng W, Yang H, Huang X, Yi L, Wen M, Jiang L, Liu F (2022). Lung cancer distress: screening thermometer meta-analysis. BMJ Support Palliat Care..

[35] Molassiotis A, Smith JA, Bennett MI, Blackhall F, Taylor D, Zavery B, Harle A, Booton R, Rankin EM, Lloyd-Williams M, Morice AH (2010). Clinical expert guidelines for the management of cough in lung cancer: report of a UK task group on cough. Cough..

[36] Dekker J, Karchoud J, Braamse AMJ, Buiting H, Konings IRHM, van Linde ME, Schuurhuizen CSEW, Sprangers MAG, Beekman ATF, Verheul HMW (2020). Clinical management of emotions in patients with cancer: introducing the approach “emotional support and case finding”. Transl Behav Med..

[37] Caraceni A, Davies A, Poulain P, Cortés-Funes H, Panchal SJ, Fanelli G (2013). Guidelines for the management of breakthrough pain in patients with cancer. J Natl Compr Canc Netw..

[38] Fallon M, Giusti R, Aielli F, Hoskin P, Rolke R, Sharma M, Ripamonti CI, ESMO Guidelines Committee (2018). Management of cancer pain in adult patients: ESMO Clinical Practice Guidelines. Ann Oncol..

[39] Wang XS, Wang Y, Guo H, Mendoza TR, Hao XS, Cleeland CS (2004). Chinese version of the M. D. Anderson Symptom Inventory: validation and application of symptom measurement in cancer patients. Cancer..

[40] Wan C, Meng Q, Yang Z, Tu X, Feng C, Tang X, Zhang C (2008). Validation of the simplified Chinese version of EORTC QLQ-C30 from the measurements of five types of inpatients with cancer. Ann Oncol..

[41] Gadsden T, Bateman-Steel CR, Chaverot S, Ressler KA, Chee K, Redwood L, Ferson MJ (2021). Using a computerised database (REDCap) to monitor influenza vaccination coverage of healthcare workers and staff in South Eastern Sydney Local Health District. Aust Health Rev..

